# Influence of Robotic Neurorehabilitation in Cerebral Palsy on Motor Function and Gait

**DOI:** 10.3390/children12020190

**Published:** 2025-02-06

**Authors:** Hristina Colovic, Dejan Nikolic, Dragan Zlatanovic, Vesna Zivkovic, Anita Stankovic, Jasna Stojkovic, Natasa Mujovic, Sindi Mitrovic, Nevena Krstic, Natasa Radosavljevic

**Affiliations:** 1 Department for Physical Medicine and Rehabilitation, Faculty of Medicine, University of Niš, 18000 Niš, Serbia; dragan.zlatanovic@medfak.ni.ac.rs (D.Z.); vesna.zivkovic@medfak.ni.ac.rs (V.Z.); anita.stankovic@medfak.ni.ac.rs (A.S.); 2 Clinic for Physical Medicine and Rehabilitation, University Clinical Center Niš, 18000 Niš, Serbia; 3 Polyclinic Neuromedic Group Plus, 18000 Niš, Serbia; 4 Faculty of Medicine, University of Belgrade, 11000 Belgrade, Serbia; dejan.nikolic@udk.bg.ac.rs (D.N.); jasna.stojkovic@med.bg.ac.rs (J.S.); natasa.mujovic@med.bg.ac.rs (N.M.); sindi.mitrovic@med.bg.ac.rs (S.M.); nevena.krstic@med.bg.ac.rs (N.K.); 5 Department of Physical Medicine and Rehabilitation, University Children’s Hospital, 11000 Belgrade, Serbia; 6 Center for Physical Medicine and Rehabilitation, University Clinical Center of Serbia, 11000 Belgrade, Serbia; 7 Clinic for Rehabilitation “Dr Miroslav Zotovic”, 11000 Belgrade, Serbia; 8 Department of Biomedical Sciences, State University of Novi Pazar, 36300 Novi Pazar, Serbia; nradosavljevic@moh.gov.sa

**Keywords:** cerebral palsy, children, rehabilitation, robotic-assisted gait training, motor function, gait

## Abstract

**Background and aim:** Cerebral palsy (CP) is a nonprogressive neurological disorder characterized by permanent developmental disorders of movement and posture. One of the most common goals of rehabilitation is the treatment of gait disorders. Ataxic gait disorder tends to worsen in the adolescent period. Research indicates a positive therapeutic effect of the combined application of conventional rehabilitation, robotic neurorehabilitation (RNR) and virtual reality, but there is no consensus on the length of treatment and frequency of application. The aim of this case report was to contribute to the definition of the RNR protocol for the treatment of ataxic gait disorder in adolescents with CP. **Case report:** In a female child with an ataxic form of CP who was on regular conventional kinesitherapy in the age period between 13 to 15 years, robotic-assisted gait training (RAGT) was applied for the treatment of gait disorders. The rehabilitation protocol lasted 10 weeks, 5 times a week, and included individual, conventional kinesitherapy for 30 min and RAGT for 30 min. Combined RNR treatment was conducted once a year in the period between July and September. The results of the therapeutic evaluation revealed that the functional motor level remained unchanged, while the improved functional motor status for the category of standing and gait was maintained during treatment between the patient’s 13 and 15 years age. In their 15th year, independent gait over a shorter distance (14 m) was achieved, as well as a normal gait frequency (83 steps/minute), with a desirable duration of the left leg support phase of 65% and 70% for the right leg support phase. **Conclusions:** The results of our research indicate that the application of conventional kinesitherapy and RAGT, over the period of 10 weeks a year, can have a positive effect on improving the postural and locomotor functions of ataxic gait in adolescents with CP.

## 1. Introduction

Cerebral palsy (CP) is considered a group of permanent movement and posture developmental disorders caused by nonprogressive cerebral lesions during fetal development and in the neonatal period leading to activity restriction [1]. With a prevalence of 2–3 per 1000 live births, CP is the most common cause of motor disability in childhood [2]. According to the type of motor deficit, there is a spastic, dyskinetic, ataxic and mixed form of CP. The ataxic form of CP is the rarest and occurs in 5–10% of children with CP. It is characterized by an abnormal pattern of posture and/or movement, as well abnormal force, rhythm and accuracy [3]. The cause is underdevelopment or abnormalities of the cerebellum, which makes it difficult to integrate neural data into the cerebellum which are necessary to control movement, balance and gait [4]. These disorders lead to significant motor deficits that limit the participation of children and adolescents with CP in activities of daily living (ADLs) and social interactions, resulting in a quality of life (QoL) decrease [5]. Many children with CP walk with non-typical gait patterns, which are often deemed problematic [6]. This is why independent gait and gait quality are the most common goal of rehabilitation among children with CP [7].

Robotic neurorehabilitation (RNR) and virtual reality stimulate the restoration of traumatized neurons over time and the reorganization of neural connections, providing an interactive interface that simulates real-life situations with the physical support of impaired motor functions [8]. Rehabilitation with a combination of traditional individual kinesitherapy, RNR and virtual reality allows for the best possible result [9]. However, the results demonstrating the effectiveness of RNR in children are still scarce, and the evidence is lacking and weak [10]. The reason is the lack of randomized controlled trials and the evident diversity in rehabilitation protocols, their duration and recommendations for the frequency of application [11,12].

We aim to present a case report that will contribute to the further definition of the RNR protocol for the treatment of ataxic gait disorder in adolescents with CP.

## 2. Case Report

A 15-year-old girl, born as a second child (older sister healthy), by caesarean section at 38 weeks of gestation with an Apgar score 10. From the age of 4 months, due to hypotonia, the patient underwent habilitation treatment. The patient started walking independently at the age of 3. Her parents reported that she walked independently in the house and with the constant presence of another person outside the house, but had a tendency and fear of falling. The diagnosis of ataxic CP was made at the age of 4. Nuclear magnetic resonance (NMR) imaging revealed moderate diffuse hypoplasia and cortico-subcortical atrophy of the cerebellum as well as asymmetrical partial ventriculomegaly of the lateral ventricles. At 10 years of age, the child could walk by holding onto furniture or another person, but she had to use a wheelchair for long distances. Prior to beginning combined RNR treatment, the child was on conventional kinesitherapy with Kabat techniques that promoted balance, carried out 4–5 times a year for 10–20 days, as well as daily exercise at home.

Prior to the first combined RNR treatment, the patient was tested for the presence of cognitive dysfunction, and non-dysfunction was noticed. The combined RNR rehabilitation protocol lasted 10 weeks, 5 times a week, and included individual, conventional kinesitherapy (stretching and muscle strengthening exercises, movement facilitation, as well as balance and positioning exercises) for 30 min and robotic-assisted gait training (RAGT) for 30 min. It was conducted once a year at the age of 13–15 years.

RAGT is performed on the LEXO^®^ gait trainer (Tyromotion, Graz, Austria) [13]. LEXO^®^ is a high-tech end-effector system for robotic gait rehabilitation that allows for free movement of the joints of the lower extremities with multiple levels of body weight support and PELVIS PLUS guidance. Gait training is active–passive with a physiological gait pattern (initial contact, stance phase, swing phase) supported by a virtual environment with visual feedback on the active participation of the lower extremities.

The observed parameters of therapeutic evaluation are functional motor level, functional motor status, gait parameters and gait cycle analysis.

To assess the achieved motor functional level, an expanded and revised Gross Motor Function Classification System (GMFCS-E&R) [14] was applied for children with CP aged from 12 to 18 years.

The gross motor function measurement-88 (GMFM-88) was used to assess functional motor status [15]. The GMFM-88 consists of 88 tasks, divided into five categories (lying and turning—A, sitting—B, crawling and kneeling—C, standing—D, and gait, running, and jumping—E) [15]. Each task is scored on a Likert scale between 0 and 3. It has been validated in children with CP up to 16 years of age. According to the goal of the study, the overall score of the GMFM-88 or an individual score for each category can be tracked. In our study, we assessed dimensions (standing abilities—GMFM-88 (D) score and GMFM-88 (E) score that considered gait, running and jumping abilities). The result is expressed as a percentage of completed tasks.

**Figure 1:**
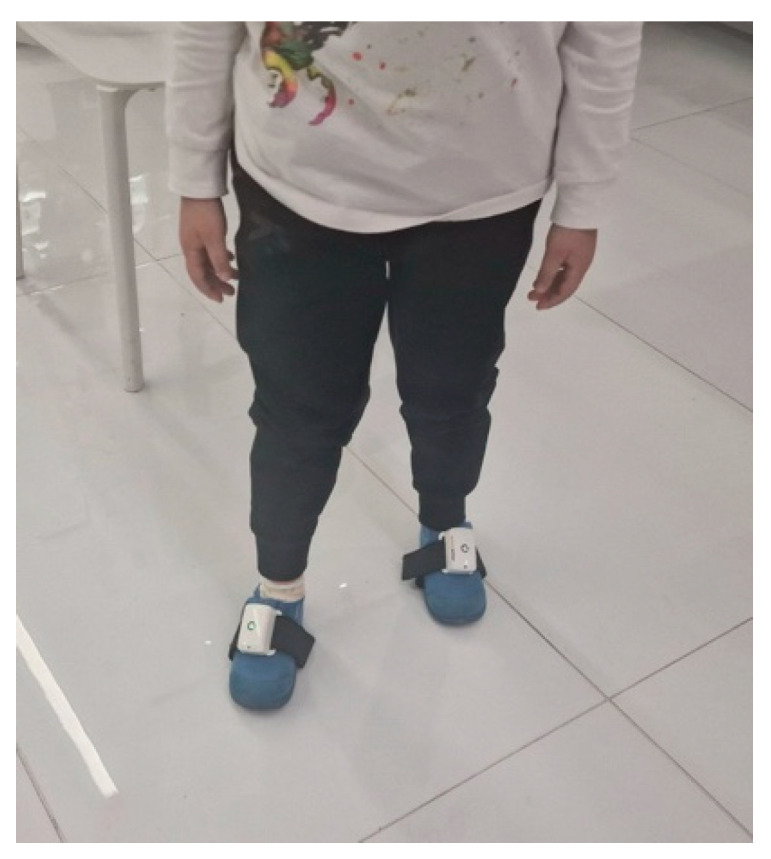
PABLO^®^ Lower Extremity.

In the evaluation of gait parameters and the analysis of the gait cycle, PABLO^®^ Lower Extremity (Tyromotion, Graz, Austria) [16] was used. PABLO^®^ Lower Extremity is an inertial measurement unit (IMU)-based gait analysis system. Wearable IMUs have the potential to enable accurate gait assessment with different pathological gait patterns. Lightweight, wirelessly portable, it does not restrict movements of the legs and the patient’s gait (Figure 1). The monitored parameters of the gate are speed (m/s), frequency (steps/min) and length of double step (cm). The analysis of the gate cycle is performed by determining the duration of the gait phases (support phase and swing phase) and is expressed in % duration during one step.

Measurements of these parameters were performed before the combined RNR treatment protocol began and on the last day of the treatment. The obtained values of the therapeutic evaluation parameters are shown in Table 1 and Table 2.

There were no adverse events during combined RNR treatment and in the measurement of outcomes.

## 3. Discussion

Independent gait is a key goal in the rehabilitation of children and adolescents with CP, as it directly affects ADL and QoL. The conventional rehabilitation protocol includes kinesitherapy with stretching and muscle strengthening exercises, movement facilitation, and balance and positioning exercises [17,18]. However, by monitoring adolescents with CP at the transition to adulthood, functional decline and reduced gait capacity were registered despite conventional rehabilitation [19]. For this reason, there is a logical need for additional therapies in the adolescent period with the aim of preserving and increasing gait capacity. De Luca et al. [20], in their study, indicated that the combined application of conventional rehabilitation and RAGT can be considered useful in improving gross motor function in children with the ataxic spastic form of CP in the domain of gait. RAGT stimulates brain neuroplasticity through the principles of motor learning via repetitive, intense exercises with the same algorithm and involving the active participation of the patient, which leads to an improvement in functional status [21].

In designing the rehabilitation protocol, we followed the current recommendations considering the patient’s age and the long-term application of conventional rehabilitation with the loss of the ability to walk independently [17,18,19,20,21]. In the selection of treatment evaluation parameters, we followed previous studies which evaluated the effects of RAGT on the improvement in gait function in children with CP, resulting in the selection of gait parameters (gait speed, frequency, double stride length) and gross motor function (GMFM-88 dimensions D and E) [22,23,24]. These studies have shown an overall improvement in all of these measurement indices [22,23,24].

However, the specificity of the ataxic form of CP is a disorder of balance control during the gait period. The work of Wallard et al. [22] is the first study, although involving a limited number of patients, to indicate a positive effect of RAGT on the control of dynamic balance during the gait period in children with CP. Analysis of locomotor parameters (gait parameters and postural stability parameters) showed that children with CP treated with RAGT adopt a new gait organization with improved postural and locomotor functions. The parameters of postural stability are the GMFM test and the duration of single and double support. Therefore, in order to assess dynamic balance, we have included an analysis of the duration of the gait phases (support phase and swing phase) for both legs.

In the current literature, there is still no consensus on the optimal parameters of RAGT in children with CP, such as duration, intensity and levels of support for the patient’s weight [25]. For this reason, in designing the rehabilitation protocol, we relied on the recommendations of the Royal College of Paediatrics and Child Health [26] for rehabilitation after pediatric stroke, which is one of the causes of CP. It is recommended to consider intensive training as well as daily treatment for at least 45 min, as long as children are willing and able to cooperate; as a result, measurable parameters improve.

The rehabilitation protocol in our case report lasted a total of 60 min (30 min of conventional rehabilitation and 30 min of RAGT), 5 times a week, for 10 weeks. In a study by De Luca et al. [20], a statistically significant effect of RAGT in children with ataxic spastic CP was achieved after 20 treatments (2–3 times a week). In a study by Wallard et al. [22], the positive effect of RAGT on the control of dynamic balance during gait in children with CP was achieved after 20 daily treatments. The average age of the children in both studies was younger, 8.6 years [20] and 8.3 years [22], while our study was conducted in a sensitive, adolescent period. In our study, the evaluation of parameters at 2 weeks pointed to a trend of improvement, with the child’s motivation during each cycle of treatment changing. During all three cycles of combined RNR treatment, stagnation of parameter values occurred between the 8th and 10th week of therapy, which corresponded to the loss of the child’s interest in further treatment.

At the age of 13, 14 and 15 years, respectively, the same rehabilitation protocol was applied once a year during school holidays (July–September). At the age of 13, at the first examination, the child was able to perform two to three steps by holding on to both hands of another person. The GMFCS-E&R was level III, the GMFM-88 (D) was 51%, and the GMFMS-88 (E) was 11%. Gait parameters and gait cycle analysis could not be examined at the start of the combined RNR treatment. The timed rehabilitation protocol was carried out in full duration, with breaks during the therapy in case of fatigue. Weight relief at the beginning of the combined RNR treatment was 50%, with a gradual decrease to 0 during therapy sessions. Due to a cold infection, the child abstained from treatment 6 times.

At the age of 14, due to family reasons, the child was absent for 4 days during the treatment, while at 15 years of age, the combined RNR treatment was carried out without any absence during one cycle. The GMFCS-E&R level III remained unchanged throughout the monitoring period. Improvement in functional motor status was recorded in both categories, GMFM-88 (D) and GMFM-88 (E), after each combined RNR treatment. The achieved GMFM-88 test value for D and E domains was maintained with the intermittent use of conventional rehabilitation between combined RNR treatments for a period of 9 months.

At the age of 15, after the third cycle of combined RNR treatment, the analysis of gait parameters indicated that desirable gait values were achieved, with 83 steps/minute.

Furthermore, gait is conditioned by the ability to produce and control the propulsive forces of the dynamic trajectory of center of mass (COM) and center of pressure (COP). Children with CP during gait must control the imbalance caused by the separation between the projection of COM and COP [22]. Dynamic balance during gait is assessed by the postural stability index (GMFM) and the duration of the support phases. On the last measurement, after combined RNR treatment at the age of 15 years of life, it was evident that the desirable duration of the support phases (left leg 65%, right leg 70%) was achieved, serving as an indicator of dynamic balance. All previous measurements had a prolonged phase of support for both legs with a slower gait, indicating a disorder of balance and gait. The highest achieved GMFM-88 (E) score of 45% was also recorded on the last test of combined RNR treatment. The length of the child’s independent walk at 15 years of age was 14 m, but for longer movements, the child still uses a wheelchair. On an uneven surface, the child needs support (walker) and it is difficult for her to move up stairs, but she has significantly improved independence in the home environment, so the GMFCS level III has remained unchanged.

## 4. Conclusions

The use of RAGT in children with CP can lead to the regaining of functional gait through intensive and repetitive simulation of different gait phases. The use of conventional rehabilitation and RAGT has a positive effect on the recovery and improvement in postural and locomotor functions of ataxic gait in children with CP during the demanding adolescent period. The lack of current research [11,20,22,23,24] explains the absence of achieved therapeutic effects of RAGT over a longer period of time, resulting in a consequent lack of recommendations and consensus on the frequency of use. A follow-up period of three years in our case report suggests that RAGT as an additional therapy to conventional rehabilitation once a year during the adolescent period enabled our CP patient to maintain her functional status, with continuous improvement during each cycle of combined RNR treatment.

## Figures and Tables

**Table 1:** Gait parameters and phase duration before and after treatment regarding age and 10 m gait performance.

Combined RNR Treatment Time	Age (Years)	10 m Walk	Gait Parameters			Gait Phase Duration (%)			
			Speed (m/s)	Frequency (Steps/Min)	Double Step Length (cm)	Support		Swing	
						L	R	L	R
Before therapy	13	Did not perform	/	/	/	/	/	/	/
After therapy		Independently	0.5	56	28	81	89	19	11
Before therapy	14	Therapist assisted	1.5	63	78	75	77	25	23
After therapy		Independently	0.6	50	39	89	90	11	10
Before therapy	15	Therapist assisted	0.6	48	45	89	88	11	12
After therapy		Independently	2.7	83	109	65	70	35	30

RNR—robotic neurorehabilitation; L—left leg, R—right leg.

**Table 2:** Standing, gait, running and jumping gross motor function measurement before and after treatment regarding age.

Age (Years)	Combined RNR Treatment Time	GMFM-88 (D) (%)	GMFM-88 (E) (%)
13 years	Before therapy	51	11
	After therapy	71	29
14 years	Before therapy	64	23
	After therapy	78	38
15 years	Before therapy	69	33
	After therapy	81	45

RNR—robotic neurorehabilitation; GMFM-88—gross motor function measurement-88; D—standing; E—gait, running and jumping.

## Data Availability

The original contributions presented in the study are included in the article, further inquiries can be directed to the corresponding author.

## References

[1] Rosenbaum P., Paneth N., Leviton A., Goldstein M., Bax M., Damiano D., Dan B., Jacobsson B. (2007). A report: The definition and classification of cerebral palsy April 2006. Dev. Med. Child Neurol. Suppl..

[2] Himmelmann K., Uvebrant P. (2018). The panorama of cerebral palsy in Sweden part XII shows that patterns changed in the birth years 2007–2010. Acta Paediatr..

[3] Kinsner-Ovaskainen A., Lanzoni M., Delobel-Ayoub M., Ehlinger V., Arnaud C., Martin S. (2017). Surveillance of Cerebral Palsy in Europe: Development of the JRC-SCPE Central Database and Public Health Indicators.

[4] Castelli E., Fazzi E., SIMFER-SINPIA Intersociety Commission (2016). Recommendations for the rehabilitation of children with cerebral palsy. Eur. J. Phys. Rehabil. Med..

[5] Makris T., Dorstyn D., Crettenden A. (2021). Quality of life in children and adolescents with cerebral palsy: A systematic review with meta-analysis. Disabil. Rehabil..

[6] Boyer E.R., Patterson A. (2018). Gait pathology subtypes are not associated with self-reported fall frequency in children with cerebral palsy. Gait Posture.

[7] Ammann-Reiffer C., Graser J.V. (2022). Gait activities beyond gait training: Priorities in everyday life for parents and adolescents in pediatric neurorehabilitation. J. Pediatr. Rehabil. Med..

[8] Marín-Medina D.S., Arenas-Vargas P.A., Arias-Botero J.C., Gómez-Vásquez M., Jaramillo-López M.F., Gaspar-Toro J.M. (2024). New approaches to recovery after stroke. Neurol. Sci..

[9] Castelli E. (2021). The Role of Robotic Rehabilitation in Children with Neurodevelopmental Disorders. Psychiatr. Danub..

[10] Conner B.C., Remec N.M., Lerner Z.F. (2022). Is robotic gait training effective for individuals with cerebral palsy? A systematic review and meta-analysis of randomized controlled trials. Clin. Rehabil..

[11] Gonzalez A., Garcia L., Kilby J., McNair P. (2021). Robotic devices for paediatric rehabilitation: A review of design features. Biomed. Eng. Online.

[12] Colovic H., Zlatanovic D., Zivkovic V., Jankovic M., Radosavljevic N., Ducic S., Ducic J., Stojkovic J., Jovanovic K., Nikolic D. (2024). A Review of Current Perspectives on Motoric Insufficiency Rehabilitation following Pediatric Stroke. Healthcare.

[13] LEXO^®^. https://tyromotion.com/en/lexo/.

[14] Palisano R.J., Rosenbaum P., Bartlett D., Livingston M.H. (2008). Content validity of the expanded and revised Gross Motor Function Classification System. Dev. Med. Child Neurol..

[15] Russell D.J., Wright M., Rosenbaum P.J., Avery L.M. (2021). Gross Motor Function Measure (GMFM-66 & GMFM-88) Users Manual.

[16] PABLO^®^. https://tyromotion.com/en/products/pablo/.

[17] Araújo P.A., Starling J.M.P., Oliveira V.C., Gontijo A.P.B., Mancini M.C. (2020). Combining balance-training interventions with other active interventions may enhance effects on postural control in children and adolescents with cerebral palsy: A systematic review and meta-analysis. Braz. J. Phys. Ther..

[18] Martin L., Baker R., Harvey A. (2010). A systematic review of common physiotherapy interventions in school-aged children with cerebral palsy. Phys. Occup. Ther. Pediatr..

[19] Morgan P., McGinley J. (2014). Gait function and decline in adults with cerebral palsy: A systematic review. Disabil. Rehabil..

[20] De Luca R., Bonanno M., Settimo C., Muratore R., Calabrò R.S. (2022). Improvement of Gait after Robotic-Assisted Training in Children with Cerebral Palsy: Are We Heading in the Right Direction?. Med. Sci..

[21] Barbeau H. (2003). Locomotor Training in Neurorehabilitation: Emerging Rehabilitation concepts. Neurorehabil. Neural. Repair..

[22] Wallard L., Dietrich G., Kerlirzin Y., Bredin J. (2018). Effect of robotic-assisted gait rehabilitation on dynamic equilibrium control in the gait of children with cerebral palsy. Gait Posture.

[23] Peri E., Turconi A.C., Biffi E., Maghini C., Panzeri D., Morganti R., Pedrocchi A., Gagliardi C. (2017). Effects of dose and duration of Robot-assisted gait training on gait ability of children affected by cerebral palsy. Technol. Health Care.

[24] Yazıcı M., Livanelioğlu A., Gücüyener K., Tekin L., Sümer E., Yakut Y. (2019). Effects of robotic rehabilitation on gait and balance in pediatric patients with hemiparetic cerebral palsy. Gait Posture.

[25] Błażkiewicz M., Hadamus A. (2024). Assessing the Efficacy of Lokomat Training in Pediatric Physiotherapy for Cerebral Palsy: A Progress Evaluation. J. Clin. Med..

[26] Royal College of Paediatrics and Child Health (2017). Stroke in Childhood: Clinical Guideline for Diagnosis, Management and Rehabilitation.

